# Bilateral Exudative Retinal Detachment in Preeclampsia: A Case Report and Literature Review

**DOI:** 10.7759/cureus.60866

**Published:** 2024-05-22

**Authors:** Sulaiman Alsamaan, Abdulmajeed Alkhathami, Azam I Alromaih, Amaal Almalki, Abdulaziz S Alsuhibani, Amani A Al Ghramah, Mohammed Nabeel

**Affiliations:** 1 Department of Ophthalmology, National Guard Health Affairs, Riyadh, SAU; 2 Department of Ophthalmology, College of Medicine, University of Bisha, Bisha, SAU; 3 Department of Medicine, King Abdulaziz Medical City Riyadh, Riyadh, SAU; 4 Department of Ophthalmology, King Abdulaziz Specialist Hospital, Taif, SAU; 5 Department of Medicine, College of Medicine, King Saud Bin Abdulaziz University for Health Sciences, Riyadh, SAU; 6 Department of Optometry, Prince Mishari Bin Saud Hospital, Baljurashi, SAU

**Keywords:** review of the literature., case report, bilateral, preeclampsia, exudative retinal detachment

## Abstract

Preeclampsia, a complex multisystem disorder predominantly impacting the kidneys and liver, manifests through hypertension and organ dysfunction in expectant mothers. Preeclampsia can also cause ocular signs, but they are uncommon. Exudative retinal detachment (ERD) is one such unusual but dangerous consequence. A thorough clinical description and therapy of a patient who experienced exudative retinal detachment while experiencing preeclampsia are provided in this study. A 28-year-old Saudi female, with no medical or surgical history, underwent an emergency cesarean section (CS) due to severe preeclampsia and failed induction of labor. The patient complained of painless blurry vision, with central dark spot and decreased vision starting from labor daytime. ﻿The patient was admitted to the hospital for blood pressure monitoring and further investigations. The patient was started on hydralazine intravenous (IV) and labetalol PO to control BP. ﻿The patient was delivered by cesarean section for preeclampsia with severe features after the failure of labor induction, and she had improved her vision by four weeks postpartum. Retinal detachment as a consequence of preeclampsia is conservatively managed, with a generally favorable prognosis. Previous studies have consistently emphasized the critical importance of a multidisciplinary approach that fosters collaboration between obstetricians and ophthalmologists. This collaborative strategy not only ensures comprehensive care but also facilitates early detection, timely intervention, and improved management outcomes for conditions affecting both maternal health and ophthalmic well-being during pregnancy.

## Introduction

The third most common cause of illness and mortality among mothers is preeclampsia [1]. Preeclampsia is a multisystem disorder unique to pregnancy, typically manifesting after 20 weeks of gestation, ﻿which occurs in the absence of other causes of elevated blood pressure and in combination with generalized edema, proteinuria, or both [2]. Preeclampsia, affecting approximately 2-8% of pregnancies globally, is characterized by hypertension and organ damage, most notably involving the kidneys and liver [3]. The ocular involvement in preeclampsia may present in various forms, including retinal artery spasm, retinal edema, and, in rare instances, exudative retinal detachment (ERD). The latter, characterized by the accumulation of fluid in the subretinal space, causing the detachment of the neurosensory retina from the retinal pigment epithelium, characterizes this condition. This separation can result in visual disturbances and potential vision loss if left untreated. It's imperative to promptly diagnose and manage this pathology to mitigate its impact on visual function and prevent further complications, ERD is an uncommon ocular manifestation that involves the accumulation of fluid in the subretinal space, leading to the separation of the neurosensory retina from the retinal pigment epithelium [4]. The visual system may be affected in 30-100% of patients with preeclampsia [5]. The most common visual symptom is blurred vision, and retinal arterioles narrowing is the most common ocular finding. An increased choroidal blood flow impedance may be the consequence of severe hypertension diseases during pregnancy. This disruption may result in exudative retinal detachment due to choroidal ischemia [6]. Despite its infrequency, the significance of exudative retinal detachment in the context of preeclampsia lies in the potential for irreversible vision loss if not managed promptly and effectively.

This study aimed to shed light on the occurrence of exudative retinal detachment in a pregnant patient with severe preeclampsia, detailing the clinical presentation, diagnostic workup, and multidisciplinary management approach that ultimately led to a favorable outcome for both mother and child. Through the exploration of this case, we hope to contribute to the existing body of knowledge surrounding the ocular complications of preeclampsia, emphasizing the importance of heightened clinical awareness and collaborative care in mitigating the impact of such rare yet critical conditions during pregnancy.

## Case presentation

A 28-year-old Saudi female, with no significant medical or surgical history, underwent an emergent cesarean section (CS) due to preeclampsia with severe features and failed induction of labor. She presented with painless blurry vision, a black scotoma, and decreased vision starting from daytime labor. Notably, there were no reported neurological symptoms such as headache, which are commonly associated with pre-eclampsia. This absence of additional neurological manifestations highlights the atypical presentation of the condition in the present case. Currently, she is prescribed labetalol 300 mg three times a day (TID) orally (PO), nifedipine 60 mg once daily PO, and hydralazine 25 mg twice a day (BID).

At 37 weeks of gestation, she was admitted for blood pressure monitoring and further investigations, showing a blood pressure (BP) of 164/107 mmHg, urine protein of 0.47 g/dL, protein/creatinine ratio of 0.54, normal platelet count, and normal liver function tests. Hydralazine intravenous (IV) 5 mg and oral labetalol were initiated to control BP, alongside a trial of labor induction with Prostin.

**Figure 1:**
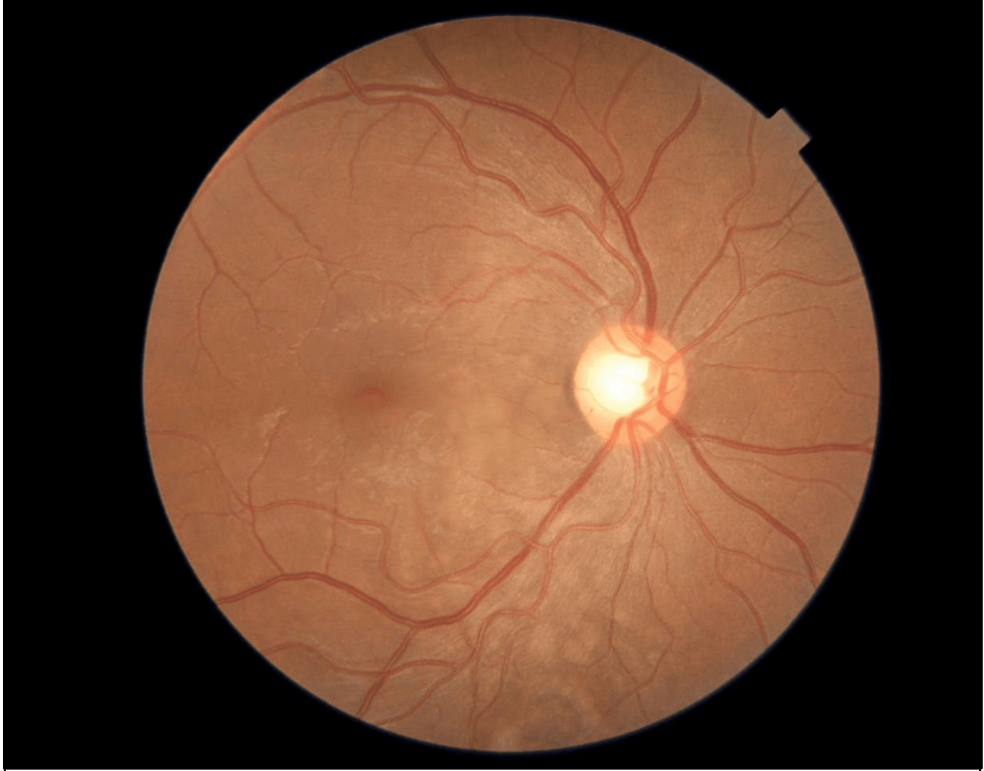
Fundus photo of the right eye showing clear media, healthy optic disc, blunt foveal reflex due to exudative retinal detachment, and normal vasculatures.

**Figure 2:**
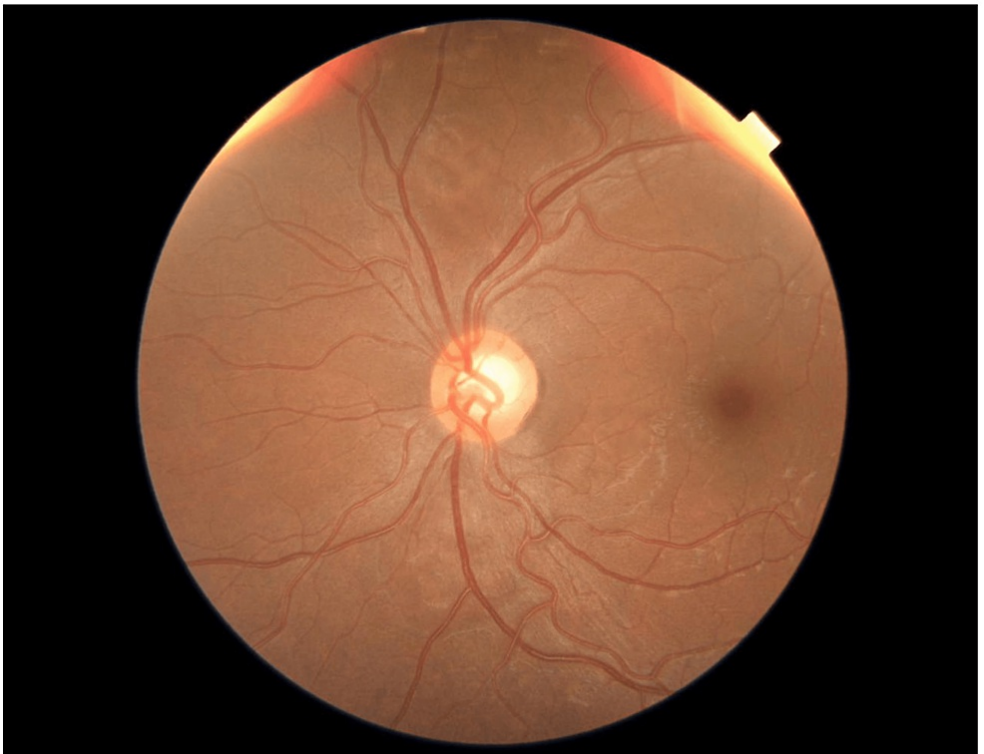
Fundus photo of the left eye showing suspected cupping of the optic disc, tortuous vessels, foveal reflex, and an epiretinal membrane.

During the cesarean section, she received four doses of Prostin, three doses of hydralazine, labetalol 300 mg, magnesium sulfate, and BP control measures. Post-delivery, her BP ranged from 150 to 160/105 mmHg, urine protein increased to 1.01 (from 0.14), and protein/creatinine ratio rose to 1.37-2.98 (from >0.3), with normal platelet count and elevated liver function tests. Physical examination findings are shown in Table 1. Slit-lamp examination findings are shown in Table 2. In Figure 1 a fundus photo of the right eye is depicted, while Figure 2 presents a fundus photo of the left eye. These images provide visual documentation of the ocular status of the patient.

**Table 1:** Physical examination findings.

Examination	Right eye (OD)	Left eye (OS)
Visual acuity without correction	20/40	20/20
Intraocular pressure (IOP)	15	16
Pupil examination	Regular, round, reactive	Regular, round, reactive
Swinging light test	No relative afferent pupillary defect	No relative afferent pupillary defect

**Table 2:** Slit-lamp examination.

Examination	OD	OS
Lid and lacrimation	Within normal limits	Within normal limits
Conjunctiva and sclera	Quiet	Quiet
Cornea	Clear	Clear
Anterior chamber	Deep and quiet	Deep and quiet
Iris	Within normal limits	Within normal limits
Lens	Clear	Clear
Dilated fundus examination	Clear view, healthy disc, multiple areas of exudative retinal detachment (ERD) in (Figure 1)	Clear view, healthy disc, small areas of exudative retinal detachment (ERD) in (Figure 2)

**Figure 3:**
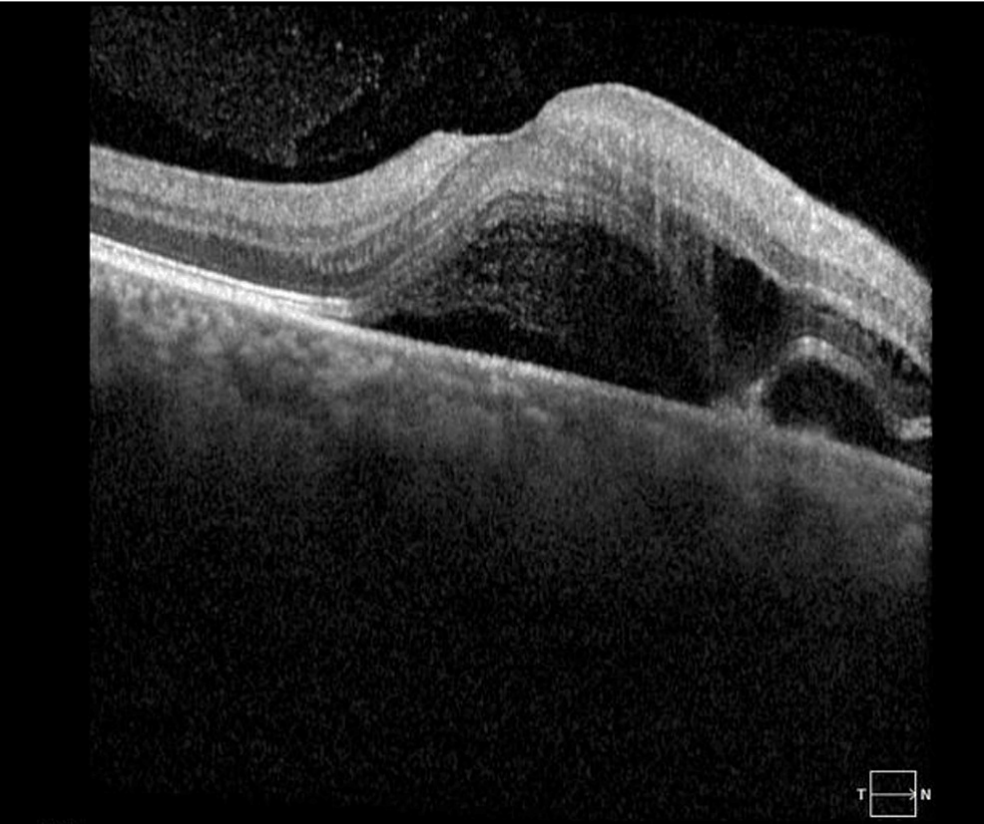
OCT of the right eye's macula reveals the central macular region exhibiting multiple areas of subretinal fluid, which involve the fovea. There is evidence of intraretinal fluid in the outer retina. The subfoveal fluid exhibits greater hyperreflectivity compared to the vitreous fluid, and multiple lobular formations of subretinal fluid are also present. OCT: optical coherence tomography

**Figure 4:**
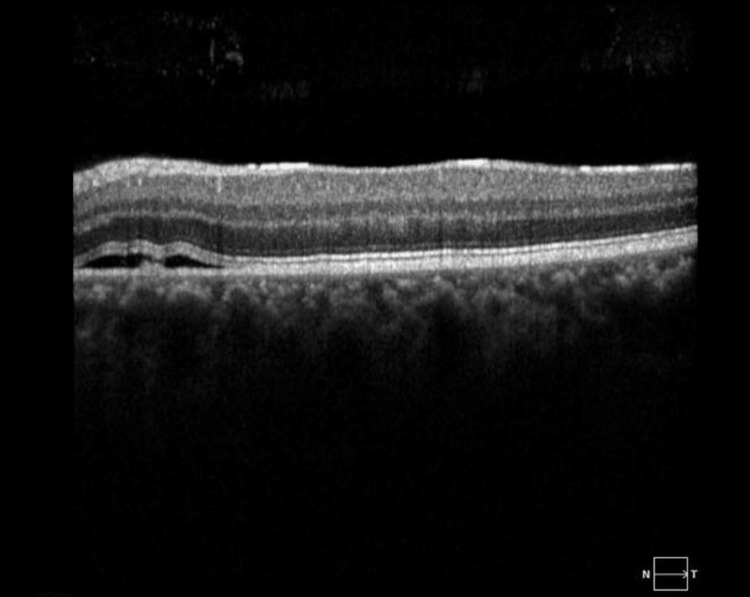
OCT of the left eye's macula, specifically a horizontal cross-section of the inferior macula, shows subretinal fluid involving the inferior nasal macula and outer retinal layers, which appear dry and flat at the center. Unlike the right eye, no subfoveal fluid is observed in the left eye. OCT: optical coherence tomography

Furthermore, Figure 3 displays the optical coherence tomography (OCT) macula of the right eye, revealing the presence of central sub-retinal fluid (SRF). This finding suggests the potential for macular edema or other retinal pathology affecting the central vision of the patient's right eye. Similarly, Figure 4 exhibits the OCT macula of the left eye, demonstrating peripheral mild SRF. This observation implies the involvement of the left eye's macula, albeit to a lesser extent compared to the right eye.

Regarding the patient's treatment and medication regimen, upon discharge, the patient reported experiencing improvement in her visual symptoms. During her hospital stay, she received appropriate medical management, including the administration of medications such as labetalol, nifedipine, and hydralazine to control her blood pressure, alongside other supportive measures.

In terms of the patient's response and recovery, it is noted that she did not attend the scheduled ophthalmology follow-up appointments. However, during subsequent visits with her primary physician, there were no complaints of visual impairment or related symptoms reported by the patient. Notably, her visual acuity returned to her baseline of 20/20 in both eyes, indicating a favorable response to the treatment provided.

Regarding follow-up care, it is imperative to emphasize the importance of regular monitoring, particularly in cases involving ocular manifestations such as those observed in this patient. Although the patient's visual acuity has returned to baseline and she remains asymptomatic, continued follow-up with both ophthalmology and primary care providers is recommended to ensure the maintenance of ocular health and overall well-being.

## Discussion

ERD in preeclampsia is an uncommon complication, as indicated by the limited number of reported cases in the literature. Previous studies have also highlighted the rarity of this condition. The low incidence may be attributed to the fact that preeclampsia itself is a relatively uncommon disorder, and the occurrence of ocular complications, such as ERD, within this context is even rare [7]. The clinical presentation of ERD in preeclampsia is consistent across studies. Patients typically present with sudden-onset visual disturbances, including blurred vision, floaters, and visual field defects [4]. Bilateral involvement is frequently observed [6]. These symptoms are similar to those seen in other causes of exudative retinal detachment and indicate SRF accumulation [8]. ERD refers to the condition where there is blood or subretinal fluid because of an acute hypertension, infection, inflammation, or tumor. The separation of the retinal pigmented epithelium (RPE) and the retinal photoreceptors as a result of this fluid buildup results in blindness [9]. The diagnostic evaluation of ERD in preeclampsia involves a combination of ophthalmic examination and imaging techniques. Fundus examination, optical coherence tomography (OCT), and B-scan ultrasonography are commonly used to confirm the presence of retinal detachment and assess the extent of the condition. These diagnostic modalities have consistently been employed in previous studies as well [10]. The exact pathophysiological mechanisms underlying ERD in preeclampsia remain incompletely understood. However, studies have proposed several contributing factors. Dysregulation of choroidal vascular permeability, endothelial dysfunction, and increased levels of vascular endothelial growth factor (VEGF) have been implicated in altering the fluid dynamics within the retina and choroid. Dysfunction of the retinal pigment epithelium (RPE) may also contribute to fluid accumulation in the subretinal space [8]. These pathophysiological mechanisms align with previous studies' hypotheses and observations. Our case report, along with previous studies, emphasizes the importance of prompt delivery in managing ERD associated with preeclampsia. Early delivery not only addresses the underlying preeclampsia but also provides an opportunity for close ophthalmic monitoring and potential resolution of retinal detachment.

Regarding long-term outcomes, the literature presents variable results. Some studies have reported complete resolution of retinal detachment and significant visual recovery following delivery, while others have noted persistent visual impairment. Factors influencing visual prognosis include the duration and severity of retinal detachment and the involvement of the macula. Unfortunately, the present study did not provide long-term follow-up data to assess the patient's visual outcome beyond the immediate postpartum period. The literature about bilateral is scanty and the majority are in the form of case reports. After reviewing the literature, we provided a narrative review of the case reports in Table 3. This comparative analysis serves to contextualize our current findings within the broader landscape of reported cases, offering insights into the variability and commonalities observed in preeclampsia.

**Table 3:** The reported case reports of exudative retinal detachment in preeclampsia patients.

Patient	Age	Gestational age	﻿﻿﻿Parity	Type of visual loss	Abnormal labs	BP	Mode of delivery	References
1	27-year-old	32nd week	Multipara	Blurred vision	Proteinuria (2.36 g in 24 h)	﻿﻿180/95 mmHg	Cesarean section (CS)	Prado et al. 2002 [3]
2	24-year-old	﻿At term of birth	﻿Primipara	Decrease in vision	Proteinuria +3, ﻿displayed leukocytosis, and a slight decrease in platelets	﻿210/140 mmHg	Normal vaginal delivery	Srećković et al. 2011 [2]
3	19-year-old	35th week	Primigravida	﻿Blurred vision in both eyes	Proteinuria +3	﻿179/102 mmHg	CS	Addenan and May 2017 [8]
4	﻿24-year-old	30th week	Primigravida	﻿New-onset headache behind ﻿the right orbit associated with central blurred vision in that eye	﻿Proteinuria	Normal (106/60 mmHg)	CS	Hussai et al. 2019 [11]
5	﻿32-year-old	33rd week	﻿Gravida III para III	Decrease in vision	None	None	CS	Khallouli et al. 2021 [6]
6	﻿25-year-old	﻿30th week	Primigravida	Total blindness upon awakening	﻿Hemoglobin concentration was 9 g/dL (standard: 12-18 g/dL), platelet count was 90 × 10^3^/µL (standard: 130-400 × 10^3^/µL), 24 h proteinuria was 6.5 g/24 h (standard <300 mg/24 h), and albuminemia was 20.34 g/L (standard: 34-54 g/L)	160/100 mmHg	CS	Zebbache 2021 [12]
7	﻿26-year-old	39th week	Primigravida	﻿Bilateral visual fog, and tinnitus	+3 proteinuria, ﻿(hemoglobin=10 g/dL, platelets=85000 plt/μL, TP=95%, lactate dehydrogenase {LDH}=880 UI/L).	185/105 mmHg	CS	Benlghazi et al. 2023 [13]

The multidisciplinary approach involving collaboration between obstetricians and ophthalmologists is consistently emphasized across previous studies. This collaborative effort ensures optimal management, timely delivery, and appropriate ophthalmic interventions, if necessary. It allows for the integration of expertise from both specialties to address the complexity of ERD in the context of preeclampsia. Given appropriate therapeutic care, the majority of patients experiencing serious retinal detachment during pregnancy recover fully within weeks of giving birth and do not require surgical intervention. Some macular changes, especially in the pigment epithelium, may persist [4,7,12,13]. This case demonstrates the excellent clinical outcome of retinal detachment in clinically controlled preeclampsia.

Limitations

As with any case report, there are inherent limitations that should be acknowledged. The present study has the following limitations: the absence of long-term follow-up restricts our ability to comment on the patient's long-term visual prognosis and the potential recurrence of ERD in subsequent pregnancies.

## Conclusions

ERD in preeclampsia is a rare ophthalmic manifestation that poses significant risks to maternal vision. While the literature on this specific condition is limited, the present study aligns with previous studies in terms of clinical presentation, diagnostic modalities, and the importance of prompt delivery. Further research is needed to elucidate the underlying pathophysiological mechanisms and assess long-term visual outcomes in patients with ERD associated with preeclampsia. Collaborative efforts between obstetricians and ophthalmologists are crucial for achieving optimal outcomes in these complex cases.
